# Evaluation of IL-3, IL-5, and IL-6 concentration in the follicular fluid of women with endometriosis: A cross-sectional study

**DOI:** 10.18502/ijrm.v20i3.10713

**Published:** 2022-04-21

**Authors:** Mohammad Ghodsi, Vida Hojati, Armin Attaranzadeh, Bita Saifi

**Affiliations:** ^1^Department of Biology, Damghan Branch, Islamic Azad University, Damghan, Iran.; ^2^Imam Reza Hospital, Mashhad University of Medical Science, Mashhad, Iran.; ^3^Department of Anatomy, Faculty of Medicine, Mashhad Medical Sciences, Islamic Azad University, Mashhad, Iran.

**Keywords:** Interleukin-3, Interleukin-5, Interleukin-6, Follicular fluid, Endometriosis.

## Abstract

**Background:**

Endometriosis is associated with abnormal immunologic responses and combined inflammatory and anti-inflammatory conditions.

**Objective:**

This study aims to investigate follicular fluid (FF) concentration of interleukin (IL)-3, IL-5, and IL-6 in women with and without endometriosis.

**Materials and Methods:**

In this cross-sectionalstudy 68 women who were referred to the in vitro fertilization center of Imam Reza hospital in Mashhad during 2018 were selected randomly. Leaves of cytokines in the FF samples were evaluated in the endometriosis and the control group (n = 34/each). The diagnostic accuracy of cytokines and clinical characteristics were evaluated.

**Results:**

IL-3 and IL-6 were significantly changed in the FF of the women with endometriosis compared with the control group (p = 0.04, and p < 0.01, respectively), and the mean concentration of IL-5 in the endometriosis group was lower than in the control group (p = 0.5), but this was not significant. There were significant differences in the menstrual cycle, dyspareunia, and dysmenorrhea between the groups (p < 0.01, p = 0.04, and p = 0.02, respectively). The diagnostic accuracy of IL-3 and IL-6 in the FF was low, with the area under the curve of 0.614 and 0.645, respectively.

**Conclusion:**

Although none of the cytokines had a predictive value for endometriosis, the decreased levels of IL-3 and increased levels of IL-6 in the FF samples of women with endometriosis, and risk factors, including irregular menstrual cycle, dyspareunia, and dysmenorrhea, could be associated with the pathogenesis of this painful disease.

## 1. Introduction

Endometriosis is a common condition in an infertile woman characterized by the growth of endometrial-like tissues with glands and stroma outside of the pelvic cavity (1). Approximately 10%-15% of women of reproductive age suffer from the disease complications related to dysmenorrhea, dyspareunia, pelvic pain, and infertility (2).

Some previous studies have demonstrated differential expression of anti-inflammatory cytokines, which play a role in the endometriosis pathogenesis (3-5). Therefore, it seems that endometriosis is a pathological condition associated with a combined inflammatory and anti-inflammatory activity where pro-inflammatory type 1 T helper (Th1) cellular response dominates the type 2 T helper (Th2) anti-inflammatory response (3, 6, 7).

Most of the previous studies have evaluated the concentration of interleukin-5 (IL-5) and IL-6 in the peritoneal fluid (PF) or serum of women with endometriosis and not in the follicular fluid (FF) (6-9). Moreover, there are conflicting results about the serum levels of IL-6 in women with endometriosis, and there are no data on the concentration of IL-3.

Hence, the present study aimed to investigate the concentration of these cytokines in the FF of women with endometriosis.

## 2. Materials and Methods

### Patient's features 

This cross-sectional study recruited a total of 68 women evaluated by laparoscopy, including 34 women with endometriosis as the endometriosis group (31.3 ± 5.6 yr old), and 34 healthy women as a control group (30.1 ± 5.5 yr old), who were referred to the in vitro fertilization (IVF) center of Imam Reza hospital (reproductive medicine unit) in Mashhad, Iran during April 2018. The control group was fertile women selected from couples referred to the IVF center for men's reproductive problems such as azoospermia.

The inclusion criteria for the control group were regular menstrual cycles, normal androgen levels, and not taking any medication. The exclusion criteria were immune system disorders, cancer, inflammatory disease, infectious diseases, history of pelvic surgery, and taking exogenous hormones three months pre-test. All participant's demographic and clinical characteristics comprising age, body mass index, endometrial thickness, menstrual cycle, galactorrhea, hirsutism, dyspareunia, and dysmenorrhea were collected and compared between the groups.

### Ovarian stimulation 

Ovarian stimulation was performed using a gonadotropin-releasing hormone antagonist protocol. Recombinant follicle-stimulating hormone treatment was commenced as 150-225 IU each day on day two of the menstrual cycle. Ovulation was triggered using 10,000 IU of human chorionic gonadotropin after observing at least two follicles with a diameter of 17 mm. After ovulation is stimulated, the best time to extract oocytes by performing a puncture through the vagina with the help of ultrasound is about 36-38 hr after the injection. Oocytes were collected separately (from four follicles per woman). Under short-term anesthesia, the follicles were pierced with a special needle through transvaginal ultrasound to remove the oocyte with the surrounding FF.

Response to the stimulation was confirmed using laboratory tests, and the size of follicles and endometrial growth were evaluated with sonography. FF was collected and used for the measurement of interleukin concentrations.

### Interleukin measurement

FF concentrations of IL-3 and IL-5 were assessed by human enzyme-linked immunosorbent assay (ELISA) kit (EASTBIOPHARM, Hangzhou, China), and concentration of IL-6 was measured with human IL-6 Platinum ELISA Kit (eBioscience, an Affymetrix Company, North America and Europe). IL-3 and IL-5 assays had a sensitivity of 1.02 pg/ml and 1.52 ng/L, respectively. Both tests showed intra-assay of coefficient of variability < 10% and inter-assay of coefficient of variability < 12%. IL-6 immunoassay had a sensitivity of 0.92 pg/m, the overall intra-assay coefficient of variation of 3.4%, and the overall inter-assay coefficient of 5.2%. The normal assay range for IL-3, IL-5, and IL-6 was 2-600 pg/ml, 3-900 ng/L (EASTBIOPHARM, China, Hangzhou), and 1-100 pg/ml (eBioscience, an Affymetrix Company, North America and Europe), respectively.

### Evaluation of total testosterone serum concentration

Total testosterone levels were determined by competitive immunoassay using the ADVIA Centaur kit, ADVIA Centaur XP, and ADVIA Centaur XPT systems (SIEMENS, USA). Measurement was performed in the early follicular phase (day 2-5 of the menstrual cycle) when the patient was fasting the night before.

### Ethical considerations

This study was a part of a project performed under the instructions of the Ethics Committees of the Islamic Azad University, Damghan Branch, Damghan, Iran (Code: IR.IAU.REC 14230525981001). Before sample collection, participants signed informed consent.

### Statistical analysis

Data were analyzed by statistical software Statistical Package for the Social Sciences (SPSS) version 16 (SPSS, Inc., Chicago, IL). The results were expressed as mean ± SD. The variables' normality was checked with the Kolmogorov-Smirnov test. The Mann-Whitney U test analyzed non-normal distributions. The Chi-square test was used to compare categorical variables between two groups in large samples, and in the case of small samples, Fisher's exact test was used (frequency < 20%). P < 0.05 was statistically significant. To determine the diagnostic accuracy of each cytokine, the receiver operating characteristics (ROC) curve analysis was used.

## 3. Results

In this cross-sectional study, 34 infertile women with endometriosis were included. They were undergoing IVF at Imam Reza hospital in Mashhad during April 2018. 34 healthy women were considered the control group who had male infertility problems (Table I). Table II shows the relationship between the menstrual cycle, hirsutism, galactorrhea, dysmenorrhea, and dyspareunia in each group.

**Figure 1:**
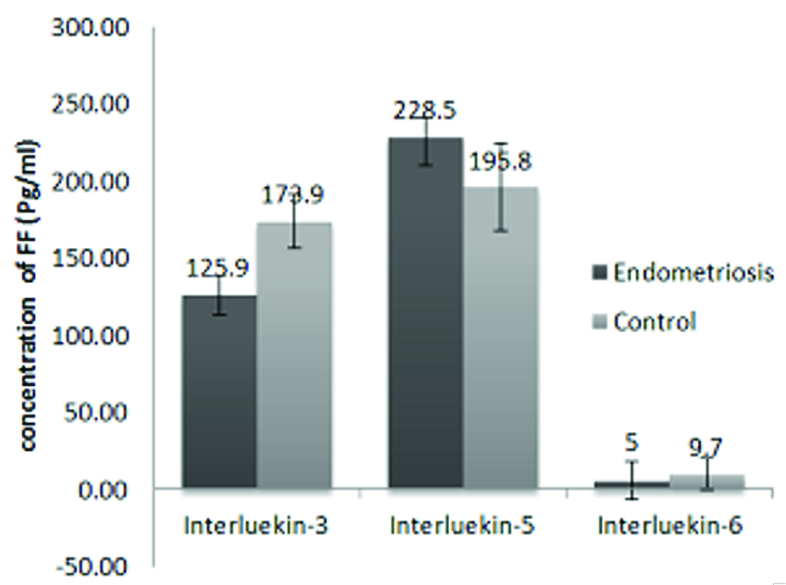
Levels of IL-3, IL-5, and IL-6 in the follicular fluids between groups. Cytokine-specific ELISA kit. Results are expressed as Mean ± SD.

FF concentrations of IL-3, IL-5, and IL-6 in women with endometriosis and controls are presented in figure 1. The mean concentration of IL-3 in the women with endometriosis was 125.9 ± 79.8 pg/ml, which was much lower than the control group (173.9 ± 109.2 pg/ml, p = 0.04). Furthermore, the mean concentration of IL-5 in the endometriosis group was lower than the control group (228.5 ± 180.7 ng/L vs. 195.8 ± 106.8 ng/L, p = 0.50). The mean concentration of IL-6 showed a significant increase in the endometriosis women (47.5 ± 72.8 pg/ml) compared to the control group (9.7 ± 6.5 pg/ml, p = 0.03).

**Figure 2:**
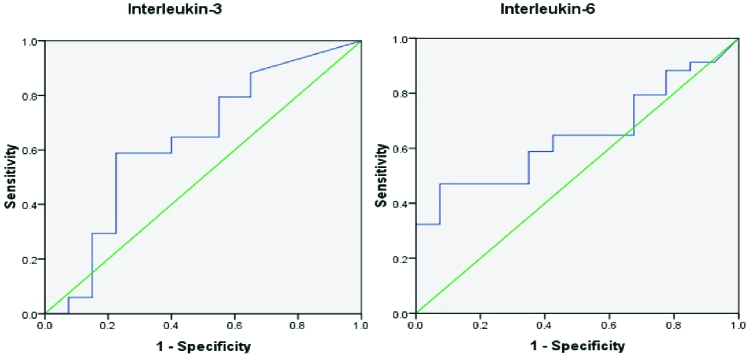
Receiver operating characteristics curve analysis shows IL-3 and IL-6 in endometriosis.

Using ROC curve analysis in the diagnosis of endometriosis, the area under the curve (AUC) for IL-3 was 0.635 (95% CI: 0.50-0.76), and the sensitivity and specificity were 58% and 60%, respectively. Furthermore, FF IL-6 achieved a low diagnostic value, with an AUC of 0.645 (95% CI: 0.51-0.78) and sensitivity and specificity of 64% and 68%, respectively (Figure 2). Table III shows the AUC, sensitivity, and specificity of IL-3, IL-5, and IL-6 for diagnosis of endometriosis with confidence intervals.

**Table 1:** Demographic characteristics in the endometriosis and control groups


**Variables**	**Endometriosis **	**Control **	**P-value**
**Age (yr)**	31.3 ± 5.6	30.1 ± 5.5	0.32^b^
**Body mass index (kg/m^2^)**	26.3 ± 4.3	25.1 ± 3.5	0.20^b^
**Endometrial thickness (mm)**	9.02 ± 2.2	9.96 ± 1.0	0.10^b^
**Testosterone levels (nd/dl)**	52.5 ± 22.3	41.9 ± 13.7	0.01*^a^
Data are presented as Mean ± SD. ^a^Based on Fisher's exact test, ^b^Based on Mann-Whitney test, *P-value < 0.05			

**Table 2:** Clinical characteristics of the control and endometriosis groups


**Variables**		**Endometriosis (n = 34)**	**Control (n = 34)**	**P** ***-*** **value**
**Menstrual cycle**				
	**Regular**	27 (79.4)	34 (100.0)	< 0.01^a^*
	**Irregular**	7 (20.6)	0	
**Galactorrhea**				
	**Negative**	33 (97.1)	34 (100.0)	0.46^b^
	**Positive**	1 (2.9)	0	
**Hirsutism**				
	**Negative**	29 (85.3)	33 (97.5)	0.09^b^
	**Positive**	5 (14.7)	1 (2.5)	
**Dyspareunia**				
	**Negative**	30 (88.2)	34 (100.0)	0.04^a^*
	**Positive**	4 (11.8)	0	
**Dysmenorrhea**				
	**Negative**	22 (64.7)	29 (87.5)	0.02^a^*
	**Positive **	12 (35.3)	5 (12.5)	
Data are presented as n (%). ^a^Based onFisher's exact test, ^b^Based on the Chi-square test, *P-value < 0.01				

**Table 3:** Sensitivity and specificity of the follicular fluid concentration of IL-3, IL-5, and IL-6


**Cytokine**	**AUC**	**95% confidence intervals of AUC**	**Sensitivity %**	**Specificity %**	**Cut-off value (pg/ml)**	**P-value**
**IL-3**	0.635	0.507-0.764	58	60	98.68	< 0.05
**IL-5**	0.456	0.317-0.594	73	43	194.16	0.51
**IL-6**	0.645	0.513-0.778	64	68	8.36	0.03
IL: Interleukin, ROC Analysis, AUC: Area under the ROC curve for ILs shows that this cytokine has a medium ability in the prediction of biochemical pregnancy						

## 4. Discussion

In this study, IL-3, IL-5, and IL-6 were measured in the FF of women with endometriosis. The results demonstrated significant alterations in the FF concentration of IL-6 and IL-3 in endometriosis women compared to the controls. Although the FF concentration of IL-5 was reduced in endometriosis women, this change was not statistically significant.

The evidence suggests that a combination of hormonal, immunological, genetic, and environmental factors is involved in the origin and development of endometriosis. Nevertheless, the etiology of endometriosis is not fully understood (3, 10). Given the crucial role of the inflammatory immune responses in the progression and development of endometriosis, the disease is considered an immune-related chronic inflammatory disease (7). Inflammation cascades lead to the activation of the immune-related cells, which produces cytokines, chemokines, and growth factors (11).

Previous findings suggested altered cytokine secretion by Th1 and Th2 cells in women with endometriosis. Changes in the balance between Th1 and Th2 cells toward Th2 may cause failure in the immunological defense system in endometriosis women (12). Altered macrophages cause ectopic endometrial cells to move into the peritoneal cavity. These cells produce inflammatory cytokines that cause the absorption and activation of T1, T2 cells (7). As stated previously, IL-6 as a pro-inflammatory cytokine contributes to the initiation and development of endometriosis via the cytokine network (9). This cytokine is predominantly secreted by macrophages. An increased number of macrophages has been reported in the PF of the women with endometriosis, which produces more IL-6, demonstrating the involvement of IL-6 in the pathogenesis of the disease (11). The present study also indicated that IL-6 concentration significantly increases in women with endometriosis. Anti-inflammatory cytokines participate in the progression of the disease through the promotion of ectopic growth, adhesion, angiogenesis, and increase in the survival of the endometrial implants (3). It seems that IL-5 elicits local inflammation in women with endometriosis and may act at the earlier stages of this inflammatory condition to develop endometrial lesions (13).

Indeed, IL-5 causes tissue damage in the inflammatory tissues. On the other hand, tissue damage induced by the growth of endometriosis activates macrophages to produce IL-6, and as a result, inflammation occurs. IL-6 produces fibrinogen and activates other coagulating factors to form thrombin at the inflammatory sites. Such a change by IL-6 helps to improve tissue damage and inhibits systemic inflammation and tissue injury. Therefore, there is an interaction between IL-5 and IL-6 so IL-6 heals IL-5-induced tissue damage.

In inflammatory diseases, such as endometriosis, IL-5 is reduced as an anti-inflammatory factor (8). The present study demonstrated no significant reduction in the FF concentration of IL-5 between the endometriosis and control groups. This discrepancy between the present study results and previous studies might be partially due to the use of different assay methods, study design, sample size, and differences in the study sample.

IL-3 and IL-5 share a beta-receptor subunit and exert similar biological activities. These cytokines regulate inflammation and are involved in the pathology of chronic inflammatory diseases (14). The current study displayed a lower concentration of IL-3 as an anti-inflammatory cytokine in the FF samples of women with endometriosis, suggesting the role of IL-3 in the pathogenesis and progression of this chronic inflammatory condition.

To our knowledge, this is the first study that assesses the FF diagnostic value of IL-3 and IL-5 to predict endometriosis in women undergoing IVF. This study indicated low diagnostic accuracy for IL-3, IL-5, and IL-6 in the FF samples based on the ROC curve analysis. None of the studied cytokines achieved a cutoff point with acceptable sensitivity and specificity.

Some studies have revealed that IL-6 could be a good marker for diagnosing endometriosis (15, 16). Jiang et al. found that the diagnostic accuracy of IL-6 in the serum and PF samples was high, with the AUC values of 0.905 and 0.952, respectively. The sensitivity and specificity of the serum IL-6 to predict the presence of endometriosis were 90% and 93.7%, respectively. Therefore, they concluded that the serum IL-6 could be used as an excellent marker to discriminate between the women with and without endometriosis (15). However, other studies in agreement with present study results reported that the predictive value of IL-6 is low and cannot be used as a suitable diagnostic or prognostic test for endometriosis (17, 18). The reasons for these conflicting and contradictory observations might be variations in the assay methods, patient selection, menstrual phases, endometriosis types and stages, and a relatively smaller sample size.

The present study showed that endometriosis is associated with irregular menstrual cycle, dysmenorrhea, and dyspareunia. These data agree with the findings of the previous studies. Hadisaputra and colleagues suggested dyspareunia and dysmenorrhea as the clinical symptoms in women with endometriosis (19). Moreover, a previous study identified a family history of endometriosis, galactorrhea history, pelvic surgery history, dysmenorrhea, pelvic pain, dyspareunia, fatigue, and diarrhea as risk factors associated with endometriosis (20).

Endometriosis can also affect the length of a person's menstrual cycles and how long the bleeding lasts. Some studies have demonstrated that short-term menstrual periods increase the risk of endometriosis (21). Since their bodies have more tissue for shedding, they may suffer from heavy menstrual cycles, which may take longer than seven days. Their cycles may also shorten, and the menstruation begins earlier than 28 days. Pain and bleeding during ovulation are the other characteristics of these women (21).

## 5. Conclusion

In summary, the results demonstrated a significant elevation in the IL-6 concentration and a remarkable reduction in the IL-3 levels in the FF samples of women with endometriosis compared to the control group, suggesting the involvement of these cytokines in the pathogenesis of endometriosis. However, the FF concentration of these cytokines offers little value in diagnosing endometriosis. Therefore, further investigations in larger sample sizes are necessary to elucidate and confirm the role of pro-and anti-inflammatory cytokines in the etiology of endometriosis and to introduce an appropriate biomarker for diagnosis of endometriosis in the early stages.

##  Conflicts of Interest

The authors declare that there is no conflict of interest.
